# Multimodal and Hyperspectral Dataset for Segmentation of Bulky Waste using VIS, IR, NIR, and Terahertz Imaging

**DOI:** 10.1038/s41597-026-07053-1

**Published:** 2026-03-27

**Authors:** Manuel Bihler, Lukas Roming, Dovilė Čibiraitė-Lukenskienė, Jochen Aderhold, Andreas Keil, Friedrich Schlüter, Robin Gruna, Michael Heizmann

**Affiliations:** 1 Karlsruhe Institute of Technology, Institute of Industrial Information Technology (IIIT), Karlsruhe, Germany; 2 Fraunhofer Institute of Optronics, System Technologies and Image Exploitation (IOSB), Karlsruhe, Germany; 3 Fraunhofer Institute for Industrial Mathematics (ITWM), Kaiserslautern, Germany; 4 Fraunhofer Institute for Wood Research, Wilhelm-Klauditz-Institut (WKI), Braunschweig, Germany

**Keywords:** Electrical and electronic engineering, Imaging and sensing, Scientific data, Information technology, Sustainability

## Abstract

This study presents an annotated multi-sensor, multimodal, and hyperspectral dataset designed to support deep learning-based classification and segmentation of bulky waste. The dataset comprises four distinct sensor modalities: high-resolution visible RGB images (VIS), hyperspectral near-infrared (NIR), temporally resolved thermal infrared (IR), and terahertz (THz) imaging with depth information, providing complementary multimodal information. An image registration process aligns all modalities to a common reference frame, enabling near pixel-precise fusion across sensors. WoodVIT contains 56 registered multi-sensor scenes, partitioned into 22,659 annotated patches with two main classes (wood and non-wood) and 16 subclass labels. It includes pixel-masks and patch-wise annotations to facilitate both segmentation and classification tasks. The primary benchmark task is binary discrimination of wood versus non-wood. The dataset also includes challenging scenarios involving occlusion and concealed contaminants (e.g., embedded metals) to motivate robust multimodal fusion approaches. We provide predefined train/validation/test splits and report baseline results using convolutional neural networks and fusion architectures to establish reference performance. WoodVIT is publicly available to support research on multi-sensor learning for waste sorting.

## Background & Summary

Waste wood recycling plays a vital role in sustainable resource management by recovering valuable materials and reducing environmental impact. In bulky waste streams, wood can represent a substantial fraction, but recycling requires reliable separation into sufficiently pure material fractions. In practice, bulky waste sorting is still often performed by hand, despite being repetitive and limited in throughput.

Sensor-based sorting provides an automated alternative by using imaging sensors to characterize material properties and trigger mechanical separation. Recent work has shown that learning-based vision systems can support bulky waste recognition using RGB imagery^1^, and that modern architectures such as Vision Transformers can be adapted via transfer learning when sufficiently large image collections are available^2^.

However, bulky waste remains challenging for single-sensor approaches due to high within-class variability and frequent occlusions. In addition, critical contaminants such as nails or screws embedded in wood may not be detectable with surface-focused modalities such as RGB or conventional infrared imaging. Recognition based on RGB alone can also be unreliable when objects are coated or painted, because surface texture features (e.g., wood grain) and color features (e.g., brown tones) may be partially or fully obscured. Complementary sensing technologies address different aspects of this problem. NIR hyperspectral imaging provides material-specific spectral signatures, especially good for distinguishing organic substances, such as plastics, including cellulose-based materials like wood, wood products and textile^3–7^. Hyperspectral imaging is already established for inline material classification of polymers and cellulose-based materials such as pulp, paper, and cardboard^3^. Typical workflows combine PCA-based dimensionality reduction with lightweight classifiers (e.g., PLS-DA, LDA, k-NN). For polymers, Zheng *et al*.^4^ showed that Fisher discriminant analysis distinguishes ABS, PS, PP, PE, PET, and PVC. In wood species discrimination, Ma *et al*.^6^ reported promising results for five softwoods and ten hardwoods using spectra within the range 1002-2130 nm. In contrast to hyperspectral imaging, active thermography probes a complementary property - the material’s transient thermal response. Low heat capacity and high emissivity materials (e.g., foams) produce strong signals while metals appear dark and it can also reveal wood grain beneath varnish^8^. Terahertz (THz) imaging offers an even higher penetration depth than thermography and exposes metallic objects concealed by non-conductive coverings, albeit at lower spatial resolution^9^.

Multi-sensor fusion for classification and segmentation is therefore a promising approach, but data-driven methods require well-annotated datasets that reflect realistic sorting conditions, and such publicly available resources remain scarce for bulky waste. Available datasets often address different application domains, simplify the problem setting, or are not multi-sensor/multimodal: remote sensing datasets can support transfer learning^10^ but do not match sensor-based sorting constraints, while many waste datasets rely on RGB only^1,11,12^ and/or focus on other waste streams such as lightweight packaging^13–16^. A dataset closer to our setting is that of Konstantinidis *et al*.^17^, which combines RGB and hyperspectral imaging (visible and NIR) but depicts clean single-object samples and is available only on request. Moreover, multi-sensor datasets that include THz measurements beyond simple metal detection are particularly rare.

To address this gap, we provide WoodVIT, a registered multi-sensor dataset for bulky waste classification and segmentation with four modalities (VIS/RGB, NIR hyperspectral, time-resolved IR, and THz with depth information). The dataset comprises 56 recorded scenes, processed into 22,659 annotated patches labeled for a two-class benchmark task (wood vs non-wood), with additional subclass labels to support detailed error analysis. WoodVIT includes challenging covered scenarios, such as embedded metal in wood, intended to support research on robust multi-sensor fusion under occlusion.

Parts of the acquisition setup have been previously described^8^, and fusion experiments are reported in more detail in^18^. The present work describes the registration, annotation, and data splitting in detail, and releases the processed data as a reusable dataset resource.

## Methods

### Experimental setting and data processing

**Fig. 1:**
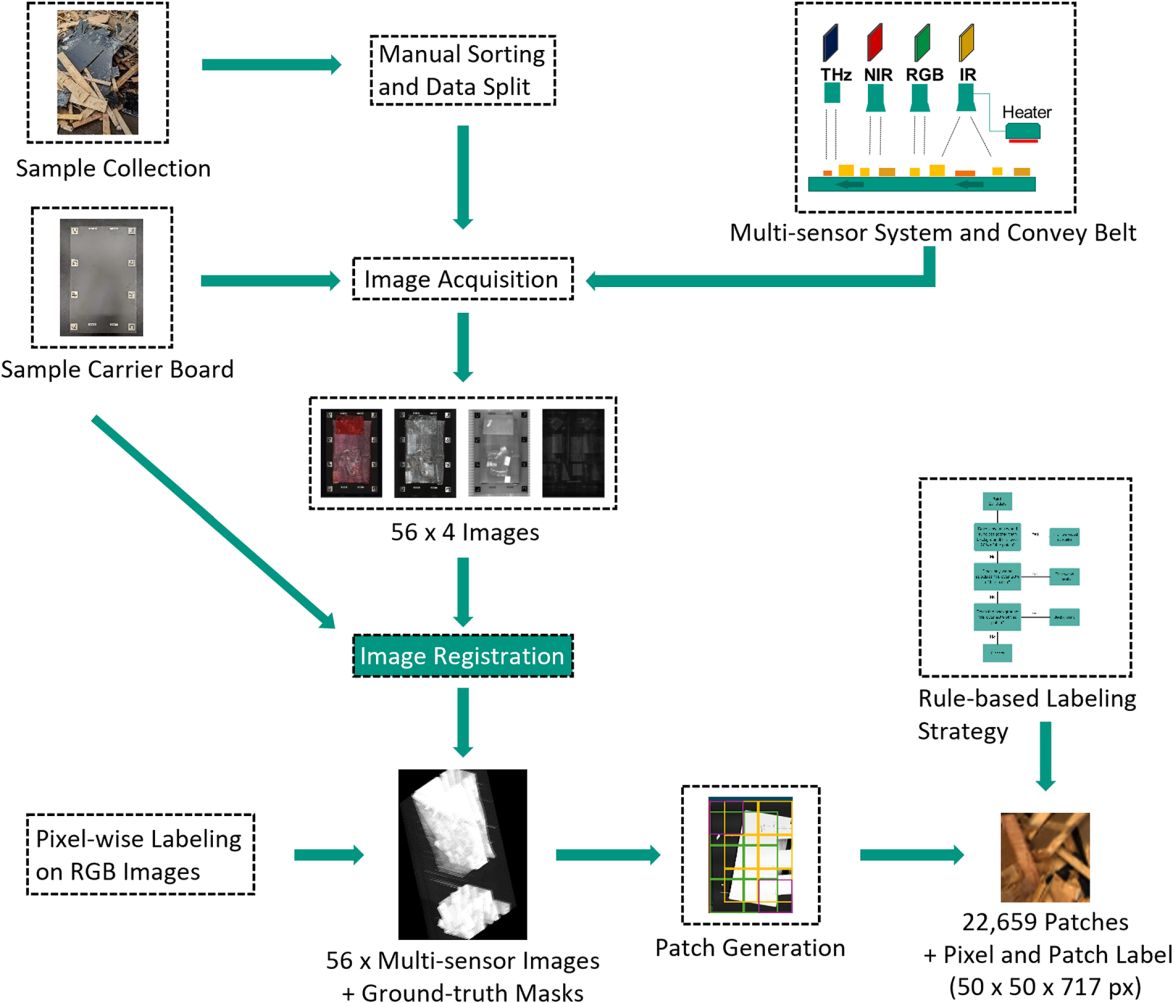
Overall scheme for acquisition and processing of the WoodVIT image dataset. Samples are sorted in 16 subclasses manually. 56 images were acquired using the previously prepared sample carrier boards and the multi-sensor system integrated with a conveyor belt. The RGB Images are labeled in 16 subclasses. All four images captured by the different sensor modalities are registered onto the corresponding RGB image, resulting in 56 registered multi-sensor images. After patch generation the dataset contains 22,659 patches with dimensions 50 × 50 × 717 px (height × width × channels), using the label criterion with the pixel-wise labels each patch receives also a patch label.

An overview of the experimental workflow is shown in Fig. 1. The primary material consisted of crushed furniture from commercially available products. Supplementary objects were obtained from a public waste-wood disposal facility in Karlsruhe, Germany.

The dataset was annotated with a hierarchical labeling scheme comprising two main classes and sixteen subclasses representing distinct materials or material combinations (Table 1). To reduce time-consuming post hoc labeling, samples were manually pre-sorted prior to imaging.

**Table 1:** Distribution of image patches across training, validation, and test subsets.

Main class	Subclass	GT index	Train	Validation	Test	Total
Wood	Solid Wood	0	2473	666	1385	4524
	Plywood	12	574	154	297	1025
	Chipboard	24	619	166	384	1169
	MDF	36	237	63	368	668
**Wood subtotal**			**3903**	**1049**	**2434**	**7386**
Non-Wood	Background	48	1556	419	903	2878
	Metal	60	1899	511	383	2793
	Plastic	72	1114	299	370	1783
	Minerals	84	483	129	297	909
	Upholstery	96	878	236	296	1410
	Wood cov. by Upholstery	108	606	163	427	1196
	Wood cov. by Cardboard	120	149	40	187	376
	Wood cov. by Plastic	132	174	46	136	356
	Wood cov. by Minerals	144	103	27	55	185
	Metal cov. by Wood	208	309	82	377	768
	Metal cov. by Upholstery	168	617	165	391	1173
	Metal cov. by Plastic	180	832	223	391	1446
**Non-Wood subtotal**			**8567**	**2340**	**4213**	**15273**
**Total**			**12470**	**3389**	**6647**	**22659**

Each subclass has a unique ground-truth index (pixel value in GT segmentation masks). Subtotals are shown per main class. Training is performed using main-class labels only. Abbreviation: cov. = covered by.

**Fig. 2:**
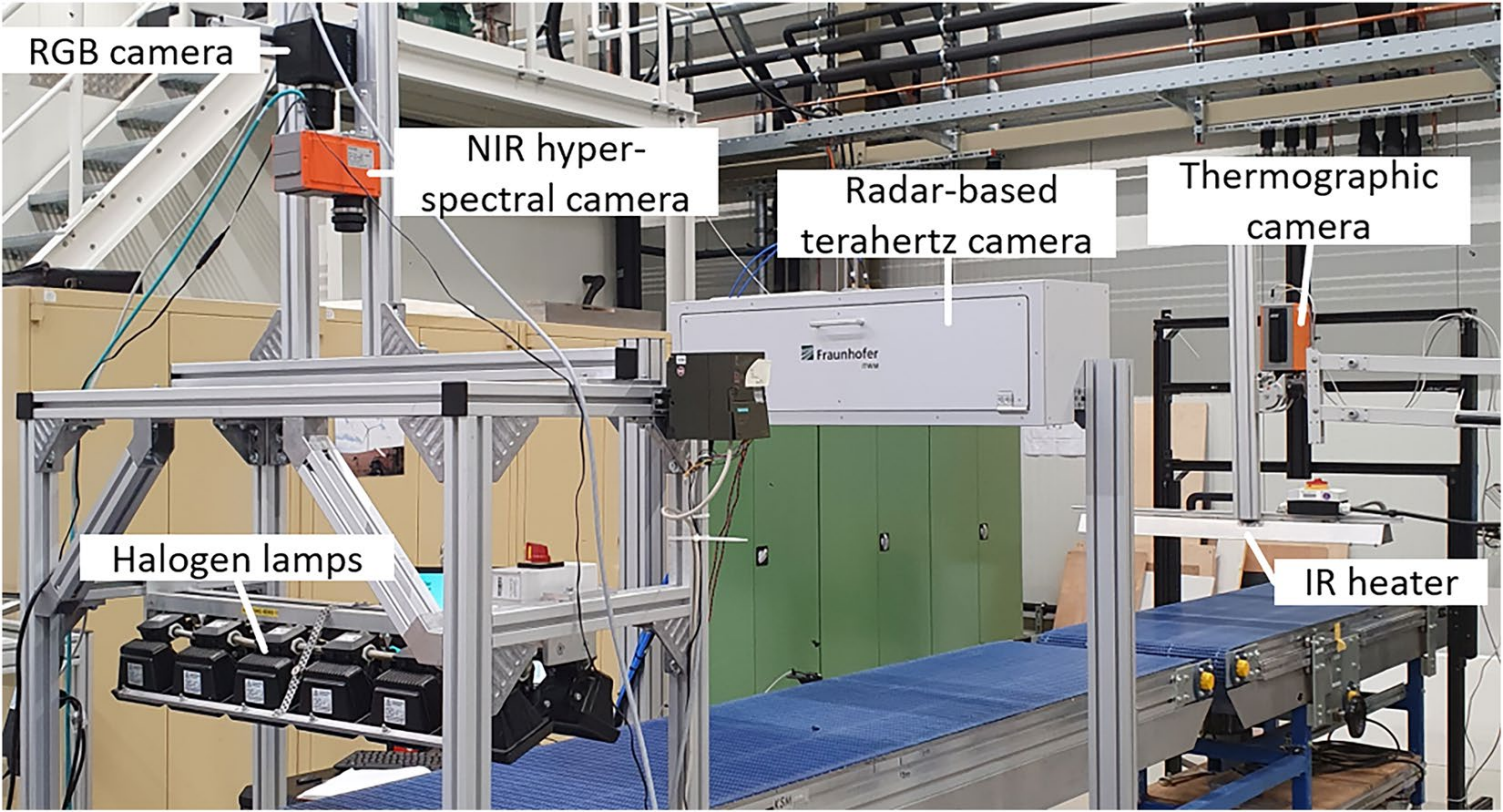
Multi-sensor measurement setup used for image acquisition. The system integrates conventional RGB, hyperspectral NIR, THz, and thermographic imaging sensors, mounted above a conveyor belt for automated sample scanning. Illumination is provided by halogen lamps and an infrared heater. Image adapted from Roming *et al*.^8^.

Multimodal data were acquired with a custom-built multi-sensor system (Fig. 2). A conveyor belt transported waste samples sequentially through four sensor stages: high-resolution RGB, hyperspectral NIR, thermography, and THz sensing.

Samples were placed on dedicated carrier boards equipped with ArUco markers^19^ and custom THz markers. These fiducials enabled reliable spatial alignment across modalities despite pronounced cross-modal heterogeneity.

During data collection, the carrier board was loaded 56 times with different pre-sorted waste samples, and one image per modality was recorded for each loading. The dataset was split at the board level into training and test subsets to ensure independence between derived patches. Images were registered using a marker-based method; pixel-wise ground-truth annotations were created on RGB and transferred to the remaining modalities via spatial registration. The four registered images were then combined into a multi-sensor image, yielding a spatially aligned stack of channels across modalities.

For downstream machine learning tasks, each multi-sensor image was subdivided into smaller patches. Patch labels were derived from the RGB ground truth using a dominant-class criterion that prioritizes high-value, low-frequency materials (e.g., metal) over more abundant but less informative classes such as wood.

### VIS/RGB data acquisition

The RGB camera captures data in the visible wavelength range and serves as the reference modality for annotation. The sensor is a prism-based RGB line scan camera (SW-4000T-10GE) with a native resolution of 4096 pixels and a selected frame rate of 625 Hz. Illumination was provided by halogen lamps. To ensure consistency across modalities, RGB images were downscaled to match the spatial resolution of the other sensors. Two-dimensional images are constructed by line scanning as samples move continuously along the conveyor belt.

### Hyperspectral NIR data acquisition

Hyperspectral imaging is a widely used technique for material characterization across various domains^20^. In this work, a line scan hyperspectral camera (FX17e, SPECIM) was used to capture near-infrared (NIR) data across 224 spectral bands in the 900 nm to 1700 nm wavelength range. The spatial resolution of the device is 640 pixels, and the frame rate was set to 104.17 Hz. Hyperspectral NIR imaging is particularly good at identifying organic materials (e.g. wood)^20^. Illumination was provided by the same halogen lamps mentioned in the RGB section, which emit in both the visible and near-infrared spectrum. Reference recordings of a white calibration target and a dark scene were used to correct for spectral sensitivity differences and spatially uneven illumination.

### Temporal thermographic data acquisition

Active thermography is a camera-based sensing technique in which samples are heated prior to imaging using an infrared heater mounted above the conveyor belt. An infrared detector records the emitted thermal radiation; the measured intensity depends on the sample temperature and its emissivity. Emissivity is a surface-specific property defined as the ratio of radiation emitted by an object to that of an ideal blackbody at the same temperature, and it is closely related to the sample’s absorptivity.

Assuming constant conveyor belt speed and stable heater properties, the observed temperature change after heating is influenced by (i) absorptivity, (ii) thermal conductivity, (iii) specific heat capacity, and (iv) mass density. By monitoring the cooling behavior with an infrared camera, it is possible to infer material characteristics related to these properties. Additionally, structured samples may develop characteristic temperature patterns, providing near-surface or subsurface features.

The infrared camera used was a Geminis 327k ML (IRCAM), equipped with a dual-band HgCdTe detector (640 × 512 px). The second sensitivity band (8 *μ*m to 9.4 *μ*m) was selected to minimize parasitic signals caused by direct irradiation from the heater. A frame rate of 625 Hz and a 25 mm lens were used. The camera was positioned so that the conveyor belt spanned the full width of the image along its longer axis. The distance between the camera and the infrared heater was 0.6 m.

### Terahertz data acquisition with depth information

**Fig. 3:**
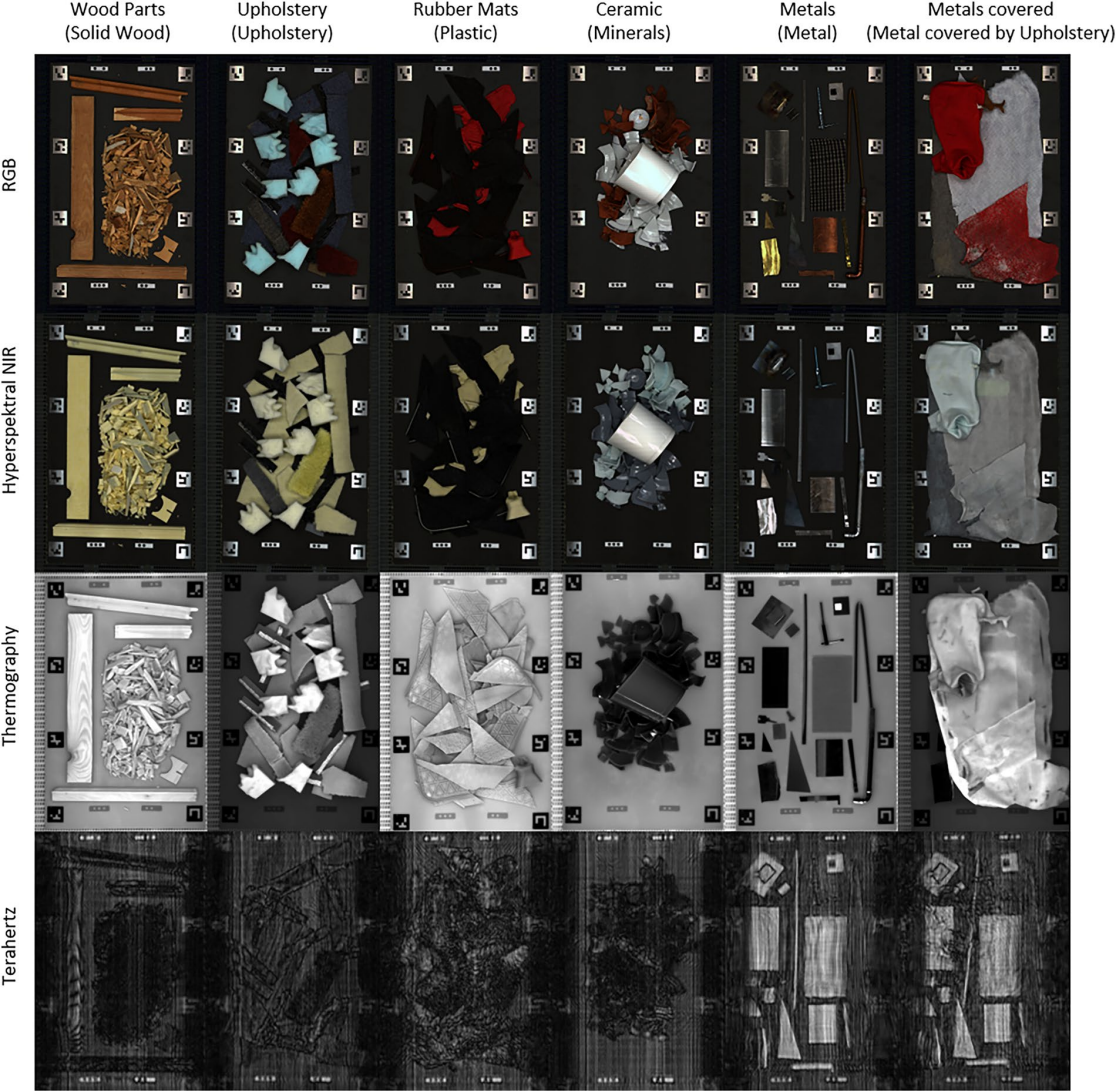
Multimodal sensor data for selected sample boards. Each row corresponds to one sensor modality (VIS/RGB, hyperspectral NIR, thermography, THz), and each column shows a different sample board representing a specific material class (indicated in brackets). The “metals covered” board contains the same metal objects as the adjacent “metals” board, but occluded by upholstery. Depth-sensitive modalities such as Terahertz and thermographic imaging enable the identification of covered or embedded materials – terahertz through subsurface penetration, and thermography through surface temperature variations that reveal differences in material structure and layering. This illustrates the potential of multimodal data to support robust classification under occluded or non-ideal conditions.

Terahertz (THz) measurements were performed using a multiple-input and multiple-output (MIMO) real-time camera operating in the 75 GHz to 110 GHz frequency range (4 mm to 2.7 mm). Most non-conductive materials are transparent to radiation in this band, making THz imaging particularly well suited for inspecting and classifying occluded materials, as conventional optical cameras primarily capture surface-level information. In total, the THz sensor returns 200 channels, each representing slices of different height relative to the sensor. Figure 3 illustrates this capability by showing a photographic image of the inspected scene alongside a representative layer of the THz image, revealing structures otherwise hidden to optical sensors.

### Image registration

To ensure spatial coherence across sensor modalities, multimodal image data must be accurately registered. This is achieved by detecting physical image markers in each image and aligning all sensor data to the corresponding RGB image from the same acquisition, which serves as the reference for annotation. Samples were imaged on a standardized carrier board equipped with eight ArUco markers and four custom THz markers. Both marker types are made of metal to enhance detectability, particularly for IR and THz modalities.

For RGB, NIR, and IR images, ArUco markers are used as reference points. In the case of RGB data, marker detection is performed directly using the OpenCV library, without the need for preprocessing. In contrast, NIR and IR modalities require data reduction to compress the multi-channel data into a single representative channel, followed by preprocessing steps such as normalization and contrast enhancement to improve marker visibility.

NIR images maintain strong spatial coherence and allow reliable marker detection using a single selected channel. Preprocessing includes normalization and inversion to account for active illumination effects. IR images, however, pose a challenge due to their construction from temporally acquired video frames rather than true line scans. Each of the 290 IR channels may exhibit minor spatial deviations due to different perspectives introduced by the temporal unfolding of the area sensor video. Therefore, marker detection is performed separately for each channel, with preprocessing steps such as normalization and contrast enhancement applied to improve detection robustness. The resulting spatial discrepancies – caused by slight perspective shifts between channels – cannot be fully corrected through marker-based alignment alone.

For THz data, the low spatial resolution prevents detection of conventional ArUco markers. Instead, four custom-designed markers with three-bit IDs are used. The THz sensor produces 200 depth slices, of which only a subset captures the markers due to their fixed position within the sensor’s field of view. Marker detection is optimized by aggregating intensity maxima across channels 160 to 190, followed by noise reduction using a median filter. Marker detection is then performed via cross-correlation with template masks, and results are validated by checking detected bit patterns. Based on the fixed geometric layout of the THz and ArUco markers on the sample board, the THz marker positions are used to infer the corresponding ArUco marker coordinates.

The final registration step uses the detected marker positions to compute a projective homography for each modality relative to the RGB image of the same sample. Sensor images are then warped using the calculated homography and concatenated. Although the sensor setup is mechanically fixed, the computed homography may vary slightly due to timing inaccuracies, belt speed variations, or physical interference during acquisition. As such, periodic homography recalibration is employed in practical scenarios to ensure consistent spatial alignment and real-time data registration.

While the homography is computed based on fixed, planar board markers, variations in sample surface height can introduce perspective distortions that cannot be corrected using a 2D transformation, leading to registration errors. These errors are typically low for RGB, NIR, and THz modalities, but can be more pronounced in the IR channels. This is due to the use of an area scan camera for temporal unwrapping in the IR acquisition, as mentioned above.

### Covered cases

To reflect industrial sorting conditions, WoodVIT includes configurations in which materials are partially or fully occluded, for example when items are stacked, wrapped, or assembled. Typical examples are nails, screws, and connectors embedded in wood, as well as furniture components where upholstery or plastics cover wooden frames and metal supports.

We exclude cases with a metal top layer because none of the included sensing modalities can reliably penetrate metal; in such configurations, the underlying material is unobservable and the task becomes ill-posed.

Ground truth for covered configurations is generated via a two-step acquisition procedure. First, the base layer (e.g., metal) is recorded alone and annotated. Subsequently, the cover layer (e.g., wood) is added on top, and the same scene is recorded again. Both acquisitions are registered using the marker-based pipeline, and the final ground-truth mask for the covered case (e.g., metal covered by wood) is obtained through a logical combination of the registered base-layer and cover-layer annotations. This procedure provides reliable labels for occluded materials, even when they are not directly visible in a given modality.

### Patch extraction

**Fig. 4:**
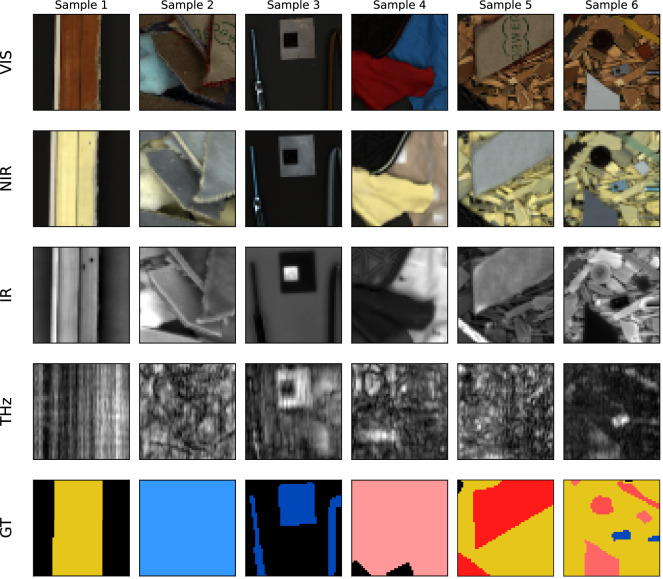
Examples of six extracted patches visualized across all sensor modalities. Each column corresponds to a different sample, and each row to one of the four sensor modalities: visible (VIS/RGB), near-infrared (NIR), infrared (IR), and Terahertz (THz), followed by the corresponding ground-truth (GT) annotation. For the multi-channel NIR, IR, and THz modalities, representative views are created using pseudo-RGB transformation, single-channel selection, and maximum-value projection, respectively.

Following registration, square patches are extracted from the registered multi-sensor images. Each patch contains 717 channels per pixel and is stored at a fixed spatial resolution of 50 × 50 px. This standardized tensor size facilitates direct use in learning-based baselines and reduces the computational cost given the 717-channel multi-sensor representation. A grid-based extraction strategy is used to maximize coverage of the sample area across all modalities. To increase variability in spatial context, three grid configurations are employed with relative patch sizes of 0.15, 0.25, and 0.35 with respect to the total image width. These correspond to physical fields of view of approximately 57 mm, 95 mm, and 133 mm on the sample carrier surface. Examples of extracted patches across all modalities, together with the corresponding ground-truth annotations, are shown in Fig. 4. The grid configurations determine the physical field of view (57-133 mm), while the fixed pixel resolution ensures consistent input dimensions across samples.

### Patch label creation

Human ground truth annotations are provided at the pixel level. To assign a class label to each patch, a rule-based labeling strategy is applied. First, we evaluate whether any non-wood subclass (excluding background) occupies more than 20% of the patch area. If so, the patch is labeled as the dominant non-wood subclass (by pixel count), enabling detection of small but relevant contaminants (e.g., metal). Otherwise, wood subclasses are assessed and the patch is labeled with the wood subclass that has the highest coverage, provided it accounts for at least 20% of the patch area. If neither wood nor non-wood subclasses meet these thresholds, background coverage is considered: patches with more than 80% background pixels are labeled as background, while all others are discarded.

### Dataset splitting

To enable fair and reproducible benchmarking, dataset splits should prevent information leakage between training and test data and avoid user-defined split ratios that hinder comparability across studies. We therefore provide a fixed training/validation/test split based on a hardware-level separation of physical samples: before acquisition, samples were sorted by subclass and assigned to two non-overlapping groups, one reserved for training/validation and one reserved for testing. The split is implemented at the board level so that all scenes from a board are assigned to the same subset, ensuring that no physical instance appears in both training and test data. This board-level separation can lead to variations in subclass frequency within the test set; in contrast, we subclass-balanced the training and validation sets to reduce variance in validation performance. Occlusion-related subclasses (all “Wood covered by ...” and “Metal covered by ...” categories) are explicitly represented in all subsets.

## Data Records

The dataset introduced in this work, referred to as WoodVIT, is publicly available in two complementary versions, both hosted on the RADAR4KIT repository:

**WoodVIT raw dataset**^21^: Available at 10.35097/yhanb6twk8t9pqku, this version contains unprocessed, unregistered sensor data acquired individually from each of the four modalities (RGB, NIR, IR, and THz). An accompanying Excel file provides metadata on measurement parameters, subclass distributions across samples, and the correspondence of sample indices between modalities. Python scripts are included to support loading and accessing the raw sensor data files.

**WoodVIT patch dataset**^22^: Available at 10.35097/aj4ve1c03pkan0dr. The patch dataset contains registered and annotated image patches, organized into training, validation, and test subsets using a hardware-level split. Each patch is stored as a NumPy array (.npy) of size 50 × 50 × 717 and is accompanied by a corresponding single-channel semantic ground-truth mask (.png). File names for both the patch data (.npy) and the masks (.png) encode patch-level metadata, including the sample identifier, the top-left pixel coordinates of the patch in the RGB reference frame, the relative extraction size, and the assigned main and subclass labels. This naming scheme enables unambiguous tracing of each patch to its spatial origin. For improved accessibility, we additionally provide a metadata table (.csv) listing these parameters for all patch data and ground-truth masks in the patch dataset.

## Data Overview

Absolute patch counts per subset after data splitting are listed in Table 1, with subclass balance maintained between the training and validation subsets. The predefined train-validation-test split is implemented at the carrier-board level to prevent overlap of physical instances across subsets.

## Technical Validation

### Registration and annotation accuracy

Accurate spatial alignment across sensor modalities is essential for pixel-level fusion and for creating reliable ground-truth (GT) annotations. Quantifying registration accuracy is challenging in our setting because the sensor modalities differ substantially in appearance, especially in the THz domain. For the same reason, automatic image-based quality checks (e.g., similarity- or feature-based alignment metrics) were not sufficiently robust across the full dataset.

To estimate the accuracy of the marker-based registration algorithm under controlled conditions, we conducted a synthetic perturbation experiment. We applied known geometric warps to the original data and subsequently re-ran the registration pipeline. The deviation between the warped marker positions and the positions estimated by the registration algorithm yields a lower-bound estimate of the registration error. Over 10 repetitions per scene, the mean deviation was 0.92 px. This experiment evaluates marker detection and warp estimation under idealized conditions. It does not capture additional error sources present in real acquisitions, such as non-linear perspective differences between modalities, height-dependent parallax for elevated objects, and other effects (e.g., conveyor motion irregularities).

For downstream machine learning, the relevant quantity is the effective alignment between sensor data and GT masks, which combines residual registration uncertainty with GT annotation uncertainty. We therefore performed a manual landmark study to estimate the total alignment error. Landmark points (*N* = 162) were defined across samples on visually identifiable structures (e.g., corners or edge intersections). Two annotators (*A* = 2) marked the corresponding location in the GT image and in each modality *s* by clicking the same structure in each image. This procedure was repeated *R* = 3 times per landmark point.

In contrast to the other modalities, the IR sensor provides a 290-frame temporal sequence whose channels are registered to each other. To assess temporal stability, we evaluated three representative frames (IR1, IR2, and IR3), corresponding to early, mid, and late time points in the sequence. We repeated the landmark-based error analysis for these frames to verify that alignment accuracy remains approximately constant over time.

For the THz modality, approximately 50% of landmark points were omitted because the structure could not be unambiguously identified in the THz image (e.g., due to limited contrast), leading to fewer valid samples for THz than for the other modalities. In total, this landmark study includes 6, 762 clicked locations ${\widehat{{\bf{x}}}}_{s,n,a,r}$.

We define the total error for modality *s* as the mean Euclidean distance between the GT landmark location and the corresponding sensor landmark location, where both locations are first averaged over the *R* repeated selections:

1 $${{\rm{Total}}{\rm{error}}}_{s}=\frac{1}{NA}\mathop{\sum }\limits_{n=1}^{N}\mathop{\sum }\limits_{a=1}^{A}\parallel \frac{1}{R}\mathop{\sum }\limits_{r=1}^{R}{\widehat{{\bf{x}}}}_{{\rm{GT}},n,a,r}-\frac{1}{R}\mathop{\sum }\limits_{r=1}^{R}{\widehat{{\bf{x}}}}_{s,n,a,r}{\parallel }_{2}.$$

**Fig. 5:**
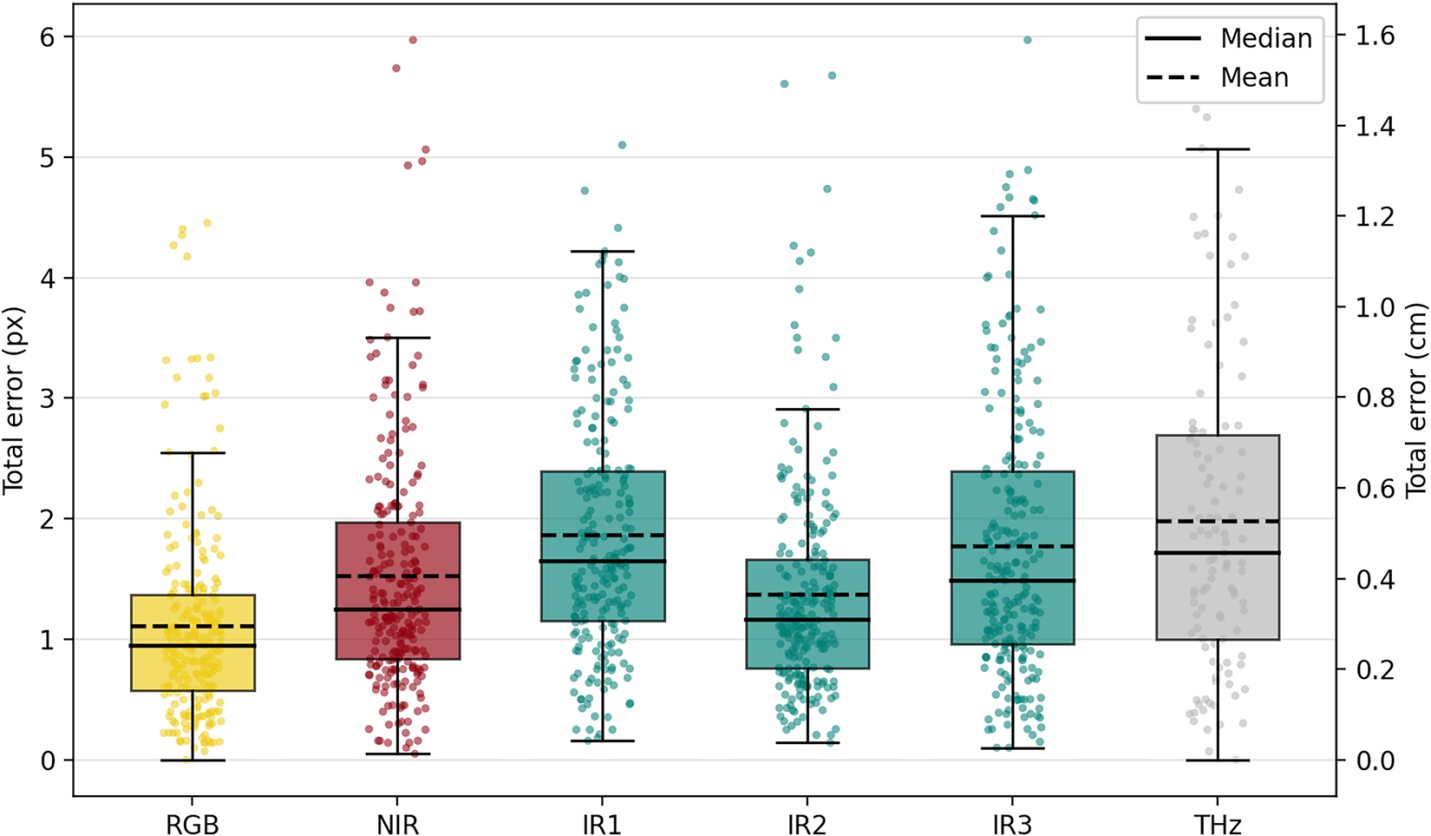
Total alignment error between each sensor modality and the GT image, estimated from *N* = 162 landmark points. Boxplots show the distribution of errors in pixels (left axis) and centimeters (right axis); solid and dashed lines indicate median and mean, respectively. IR1, IR2, and IR3 correspond to early, mid, and late frames of the time-resolved IR sequence.

The distribution of Total error_*s*_ across all sensor modalities is shown in Fig. 5, reported in pixels and additionally converted to centimeters using the known physical scale of the sample board.

Total error_*s*_ aggregates several sources of uncertainty. It includes (i) residual registration error between modalities, (ii) GT annotation error introduced during GT creation (manual annotation based on RGB images), and (iii) measurement error from the landmark study itself. For downstream machine learning, the combined alignment uncertainty between sensor data and GT is the most relevant quantity; we therefore report Total error_*s*_ as a conservative summary of alignment accuracy.

We estimate the measurement error (iii) as the per-landmark, within-annotator standard deviation across the *R* repeated selections, computed separately for each modality. Using this definition, the measurement error was 0.15 px (GT), 0.19 px (RGB), 0.20 px (NIR), 0.22 px (IR; aggregated over IR1–IR3), and 0.25 px (THz).

Second, we approximate the GT annotation error (ii) from the RGB-to-GT alignment. Because RGB defines the reference frame for registration, no cross-modal registration component is present for RGB, and Total error_RGB_ is dominated by GT annotation error and measurement error. Based on Fig. 5, the GT annotation error is approximately 1 px.

Accordingly, Total error_*s*_ can be interpreted as an upper bound on the residual registration error (i) for modality *s*. Across modalities, Total error_*s*_ is typically below 1.5 px on average (Fig. 5), indicating near pixel-level alignment in most cases. The remaining spread is primarily driven by modality-specific limitations such as reduced spatial detail in IR/THz and height-dependent parallax in non-planar scenes, where elevated objects introduce viewpoint-induced displacements relative to the planar sample board, which cannot be fully corrected by a marker-based registration model.

### Multimodal data visualization

**Fig. 6:**
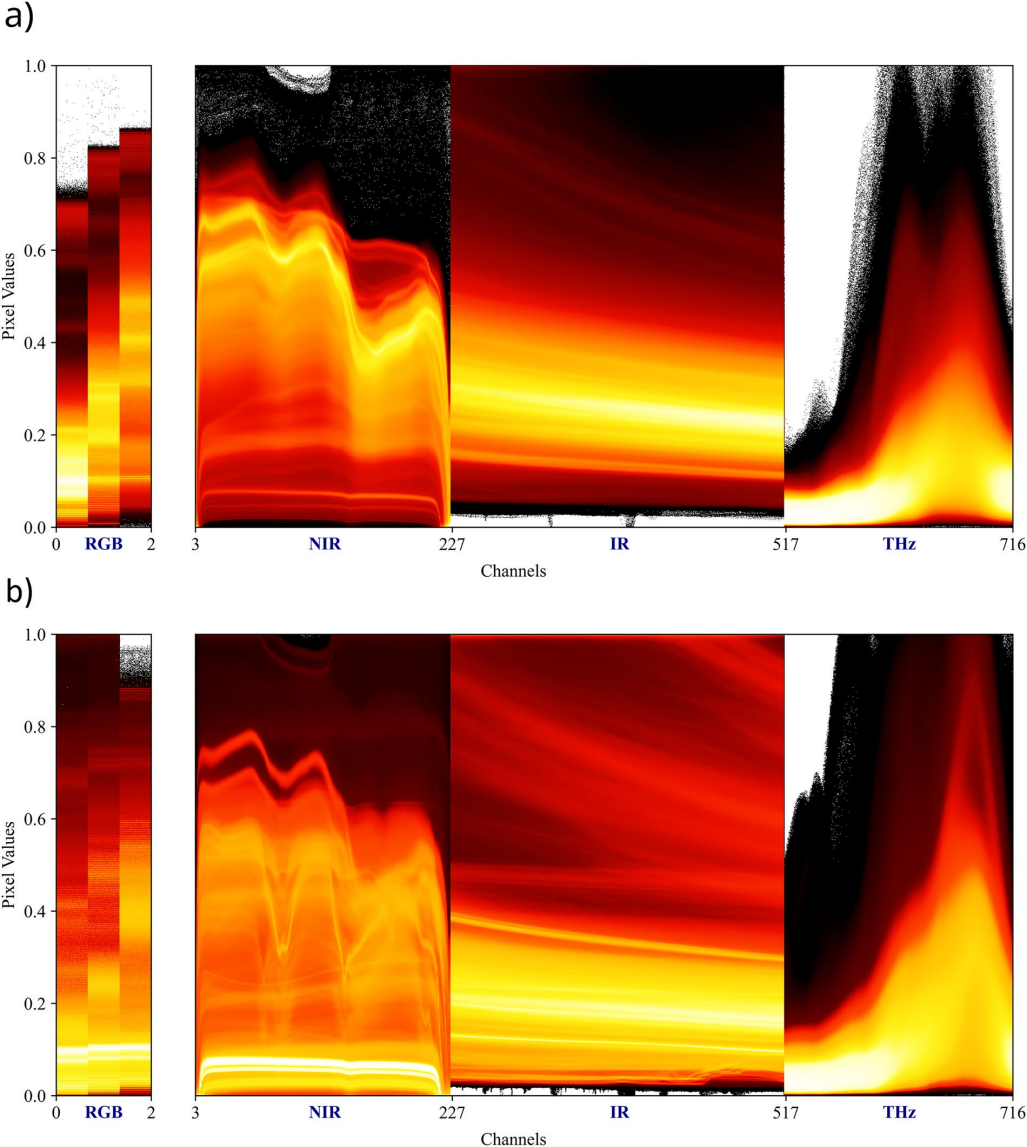
Pixel intensity distributions across all sensor channels for ‘Wood’ (a) and ‘Non-Wood’ (b) labeled data. The x-axis shows sensor channels sequentially: RGB (channels 0 to 2, expanded width for clarity), NIR (3 to 227), IR (228 to 517), and THz (518 to 716). The y-axis represents normalized pixel intensity values, with color intensity indicating pixel frequency (brighter areas correspond to higher frequency).

To provide an overview of the dynamic range and typical signal patterns across modalities, we summarize pixel intensities per sensor channel for the two main classes (Wood, Non-Wood) in Fig. 6. For each channel, pixel values from all patches of a given main class were aggregated and visualized as a normalized 2D intensity histogram.

Clear differences between the two classes are observed across modalities. The NIR data display characteristic spectral signatures, prominently reflecting the absorption and reflection properties of materials like plastics. In the IR modality, the data reveal intensity slopes typical of exponential cooling after heat exposure; notably, the ‘Wood’ class exhibits minimal variance, contrasting with the higher variability seen in the more compositionally diverse ‘Non-Wood’ class. The THz data are more diffuse overall but show concentrated pixel intensities in central channels, indicating frequent occurrences of metals at specific depths.

### Class balance

The dataset includes subclass annotations that are not used for model training. Due to insufficient variability and limited sample counts in the subclasses, these subclasses cannot be reliably represented as independent training classes without introducing poorly defined learning targets. Instead, subclass labels are provided solely as additional information to assess the generalization behavior of models trained on the main classes. Accordingly, training, class balance and all performance evaluations are done exclusively with respect to the main classes.

We quantify class imbalance using the imbalance ratio

2 $$\rho =\frac{\mathop{\max }\limits_{i}\left|{C}_{i}\right|}{\mathop{\min }\limits_{i}\left|{C}_{i}\right|},$$

as defined in^23^, where *C*_*i*_ denotes the set of examples belonging to class *i*, and $\mathop{\max }\limits_{i}\left|{C}_{i}\right|$ and $\mathop{\min }\limits_{i}\left|{C}_{i}\right|$ return the maximum and minimum class size over all *i* classes, respectively. Considering only the main classes, our dataset exhibits an imbalance ratio of

$${\rho }_{{\rm{train}}}=\frac{8567}{3903}=2.195,$$

which is comparable to the lowest imbalance settings considered in prior work (e.g., CIFAR-10 with *ρ* ≈ 2 in^23^). By contrast, imbalanced learning studies typically address much larger imbalance ratios, often exceeding one order of magnitude, as discussed in^24^.

For this mild imbalance setting, random minority oversampling is a common and effective strategy. Following this established practice, we employ random minority oversampling to ensure stable and efficient convergence of all models. The final train/validation split preserves uniform subclass proportions.

### Image classification task

To assess the suitability of the WoodVIT dataset for machine learning applications, we performed a technical evaluation using multiple convolutional neural network (CNN) architectures. Due to the limited number of representative samples per subclass, all experiments were restricted to the two main material classes.

We implemented three custom CNN models of increasing complexity, as well as SpectrumNetDSBN. The latter is a modified version of SpectrumNet^25^ that incorporates depthwise separable convolutions to improve performance on small multi-spectral datasets. Each model was trained independently using data from four individual sensor modalities (RGB, NIR, IR, and THz) as well as early-fused (EF) data^26^, where all sensor modalities are combined into a single input by channel-wise concatenation.

All experiments used a batch size of 32, a fixed learning rate of 0.0005, and 100 training epochs. CNN1BN served as the baseline model and consists of simple convolutional and fully connected layers. Subsequent model variants introduced pooling layers, depthwise separable convolutions, and global average pooling to improve feature extraction while controlling model complexity.

Data augmentation included horizontal and vertical flips as well as rotations. To preserve the integrity of the patch-level labels, which are assigned using a rule-based labeling strategy, augmentation was restricted to spatial transformations that do not alter the material composition within a patch.

Classification performance was evaluated using accuracy, precision, recall, F1-score, and the normalized Matthews Correlation Coefficient (normMCC). All metrics were derived from the confusion matrix entries true positives (TP), false positives (FP), true negatives (TN), and false negatives (FN). Accuracy is defined as

3 $${\rm{Accuracy}}=\frac{{\rm{TP}}+{\rm{TN}}}{{\rm{TP}}+{\rm{TN}}+{\rm{FP}}+{\rm{FN}}},$$

while precision and recall are given by

4 $${\rm{Precision}}=\frac{{\rm{TP}}}{{\rm{TP}}+{\rm{FP}}},\qquad {\rm{Recall}}=\frac{{\rm{TP}}}{{\rm{TP}}+{\rm{FN}}}.$$

The F1-score is computed as the harmonic mean of precision and recall,

5 $${F}_{1}=\frac{2\cdot {\rm{Precision}}\cdot {\rm{Recall}}}{{\rm{Precision}}+{\rm{Recall}}}.$$

The Matthews Correlation Coefficient (MCC) is defined as

6 $${\rm{MCC}}=\frac{{\rm{TP}}\cdot {\rm{TN}}-{\rm{FP}}\cdot {\rm{FN}}}{\sqrt{({\rm{TP}}+{\rm{FP}})({\rm{TP}}+{\rm{FN}})({\rm{TN}}+{\rm{FP}})({\rm{TN}}+{\rm{FN}})}},$$

and subsequently normalized as normMCC = (MCC + 1)/2, mapping values to the interval [0, 1]. While accuracy provides a general measure of performance, it can be insensitive to class-specific behavior in imbalanced settings. Precision, recall, and F1-score emphasize complementary error characteristics but ignore true negatives^27,28^. The use of normMCC therefore enables a more balanced assessment by incorporating all four confusion matrix entries^29^. Together, these metrics provide a robust and model-agnostic assessment of dataset suitability across different learning configurations.

**Fig. 7:**
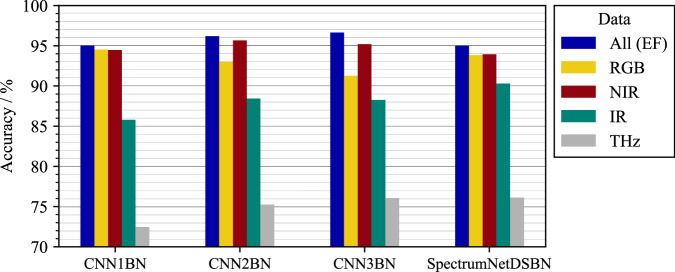
Classification results for all sensor modalities across different models in a binary classification task. The bar chart shows the accuracy of CNN1BN, CNN2BN, CNN3BN, and SpectrumNetDSBN for early-fused (EF), RGB, NIR, IR, and THz data. Early-fused data consistently show higher accuracy than individual sensor modalities, while IR and THz data exhibit lower classification performance.

All experiments were conducted as binary classification tasks, with wood defined as the positive class, reflecting the limited number of representative samples available per subclass. Figure 7 compares the performance of different models across sensor modalities.

Table 2 summarizes the performance of the CNN1BN model across different sensor inputs and fusion strategies, including early fusion and late fusion. In the early fusion approach (data-level fusion), all sensor data are concatenated prior to training and used as input to a single end-to-end model^18^. In the late fusion (decision-level fusion) approach, four separate models are first trained independently on modality-specific data. Their outputs are then combined using a multi-layer perceptron (MLP) with two hidden layers consisting of 16 and 8 neurons, respectively. To mitigate the risk of overfitting given the limited dataset size, the late-fusion model was kept small on purpose.

**Table 2:** Performance metrics for different fusion strategies in a binary classification task.

Fusion	Sensor	Accuracy	Recall	Precision	F1-Score	normMCC
None	RGB	0.91 ± 0.02	0.91 ± 0.01	0.86 ± 0.02	0.88 ± 0.01	0.90 ± 0.02
None	NIR	0.93 ± 0.02	0.97 ± 0.01	0.89 ± 0.02	0.93 ± 0.02	0.93 ± 0.02
None	IR	0.87 ± 0.02	0.88 ± 0.02	0.79 ± 0.02	0.83 ± 0.02	0.86 ± 0.02
None	THz	0.73 ± 0.03	0.62 ± 0.04	0.64 ± 0.01	0.63 ± 0.02	0.71 ± 0.03
EF	All	0.94 ± 0.00	0.96 ± 0.02	0.89 ± 0.02	0.92 ± 0.01	0.94 ± 0.01
LF	All	0.95 ± 0.01	0.91 ± 0.01	0.94 ± 0.00	0.93 ± 0.01	0.94 ± 0.01

Metrics are reported as mean  ± standard deviation over four runs with different random seed initializations. LF denotes late fusion and EF denotes early fusion. CNN1BN was chosen as the base model for all results.

Overall, the dataset presents distinct challenges across sensor modalities, with IR and THz data exhibiting lower classification performance. In contrast, both early-fused and late-fused configurations achieve slightly higher accuracies with reduced standard deviation, indicating a more stable and reliable training process. The fusion experiments presented here are intended as a technical validation of the dataset.

### Subclass usage

Although training is performed using only the two main classes (Wood and Non-Wood), we retain subclass labels for evaluation to support a more detailed interpretation of model behavior, particularly for sensor fusion. Specifically, we compute a subclass confusion matrix of size 16 × 2 that maps the 16 GT subclasses to the two predicted main classes, enabling fine-grained error analysis beyond the aggregated 2-class results.

**Fig. 8:**
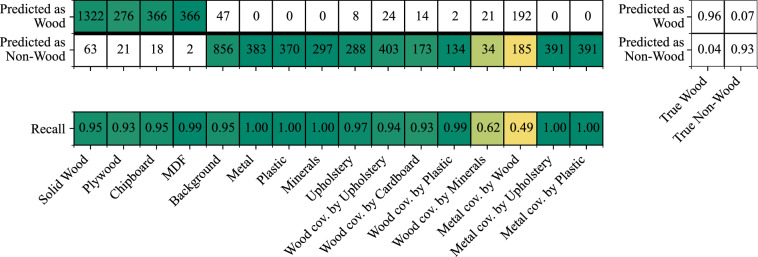
Confusion matrices and subclass-wise recall for the early-fusion model evaluated on the test set. Top left: Subclass-level confusion matrix (16 × 2) comparing GT subclass annotations (16 subclasses) to model predictions for the two main classes (Wood, Non-Wood). Absolute sample counts are shown; cell coloring indicates the assignment of each subclass to its corresponding main class, equivalent to the two diagonal blocks of a standard 2 × 2 confusion matrix. Top right: Aggregated confusion matrix reporting true positives (TP), false positives (FP), true negatives (TN), and false negatives (FN), with Wood treated as the positive class. Bottom: Recall values for all subclasses; color scales indicate recall performance across subclasses. Abbreviation: cov. = covered by.

An example is shown in Fig. 8 for an early-fusion model, where all sensor modalities are concatenated at the input level. While the model is trained only on the main classes, subclass-wise recall reveals how well the learned representation transfers across different subclasses and highlights cases that are not fully captured by this generalization.

Early-fusion models show reduced performance for the subclass Metal covered by Wood. This is consistent with modality conflict: surface modalities (RGB, NIR, IR) primarily reflect the wood layer, whereas only the THz modality provides evidence of the concealed metal. Subclass-level evaluation therefore helps diagnose fusion failure modes without introducing subclasses as training targets.

### Semantic segmentation task

The suitability of the WoodVIT dataset for semantic segmentation was examined using U-Net architectures trained from scratch on RGB and near-infrared (NIR) image modalities. RGB and NIR were selected for this experiment because they showed the most stable behavior in the preceding classification-based technical validation. Original input images of 50 × 50 pixels were resized to 256 × 256 pixels to facilitate enhanced feature extraction, harmonized downsampling to a 16 × 16 bottleneck, and compatibility with a five-level U-Net architecture.

**Fig. 9:**
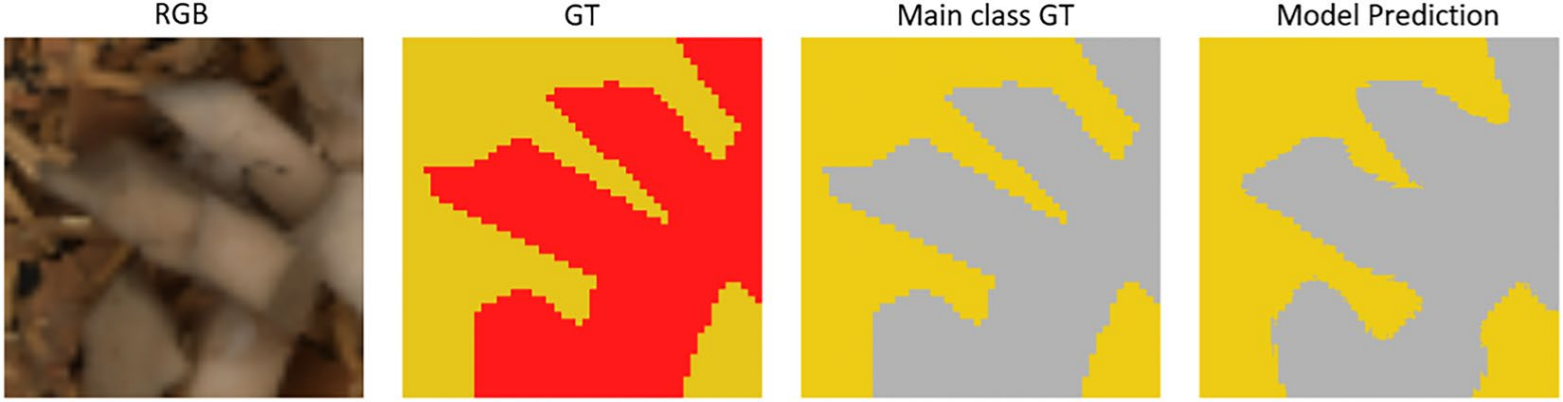
Semantic segmentation result using RGB input and a U-Net architecture. From left to right: (i) Original RGB image (50 × 50 pixels), (ii) GT mask with subclass annotations, (iii) binary GT mask used for training (yellow = wood, grey = non-wood), and (iv) model prediction. To improve performance, both the RGB image and the GT mask were upscaled to 256 × 256 pixels before training and inference.

The segmentation workflow, illustrated in Fig. 9, comprised (i) original RGB images, (ii) subclass-level GT annotations, (iii) binary training masks distinguishing wood from non-wood regions, and (iv) final model predictions. For training, subclass annotations were converted into binary masks, enabling a pixel-level wood versus non-wood segmentation task.

Both models were trained for 500 epochs using a batch size of 20 and a learning rate of 10^−4^. To address class imbalance between wood and non-wood pixels, a composite loss function combining weighted cross-entropy and Dice loss with equal weighting was employed. All segmentation experiments were repeated four times using different random seed initializations, and variability across runs is reported as standard deviation.

Segmentation performance was evaluated using Intersection over Union (IoU) and pixel-wise accuracy, defined as the ratio of correctly classified pixels (true positives and true negatives) to the total number of pixels. The RGB-based model yielded an IoU of 0.84 and a pixel-wise accuracy of 0.93, while the NIR-based model yielded an IoU of 0.85 and an accuracy of 0.92.

These results provide an initial verification of the dataset’s applicability to pixel-level semantic segmentation tasks.

## Usage Notes

The dataset has undergone preprocessing, including image registration, to enable early fusion approaches across modalities. Specifically, the RGB images were downsampled to align spatially with other modalities. Users seeking access to the original, unregistered, full-resolution data are referred to the raw dataset repository.

Additional preprocessing and augmentation may improve robustness, but should respect the rule-based patch labeling. Use label-preserving transforms (e.g., brightness/contrast changes and rotations without cropping) and avoid operations that change spatial composition (e.g., cropping or strong geometric warps), as these can invalidate patch labels.

Notable limitations of the dataset should also be considered. Minor inaccuracies in pixel-level annotations are present, particularly near object boundaries. As shown in the Technical Validation section, these inaccuracies do not substantially impact model training.

Furthermore, due to limited sample diversity, simultaneous training across all 16 classes may lead to model overfitting. Users are therefore encouraged to design their experiments thoughtfully, potentially limiting the number of classes included in initial training runs.

For fusion-specific benchmarking under occlusion, users can filter patches by these subclasses using the provided metadata table and optionally oversample them during training or evaluate on a dedicated occlusion-only subset.

The dataset is publicly available under the Creative Commons Attribution 4.0 International License (CC BY 4.0). Users must provide appropriate attribution when using the dataset.

## Data Availability

Both versions of the WoodVIT dataset are publicly available via the RADAR4KIT repository: the raw, unregistered multi-sensor recordings (10.35097/yhanb6twk8t9pqku) and the registered, annotated patch dataset used in this work (10.35097/aj4ve1c03pkan0dr). The data are released under the Creative Commons Attribution 4.0 International License (CC BY 4.0).

## Code availability

Registration and data processing were implemented in Python using *Spectral* (version 0.23.1), *Datashader*, and *OpenCV* (version 4.10.0). Model training and evaluation for the baselines reported in this work were carried out in *PyTorch*^30^ (version 2.1.2) with *CUDA* (version 12.1). We provide the baseline training and evaluation scripts, example configurations, and selected model checkpoints at: https://github.com/ASKIVIT-Project/WoodVIT-V1-training.
